# Pervasive Parental Hesitancy and Resistance towards Measles Rubella Vaccination in Jordan

**DOI:** 10.3390/vaccines11111672

**Published:** 2023-10-31

**Authors:** Muna Barakat, Maram Abdaljaleel, Nada Atawneh, Rawan Alkhazaleh, Dana Aburumman, Eman Hamed, Malik Sallam

**Affiliations:** 1 Department of Clinical Pharmacy and Therapeutics, Faculty of Pharmacy, Applied Science Private University, Amman 11931, Jordan; m_barakat@asu.edu.jo; 2 MEU Research Unit, Middle East University, Amman 11831, Jordan; 3 Department of Pathology, Microbiology and Forensic Medicine, School of Medicine, The University of Jordan, Amman 11942, Jordan; 4 Department of Clinical Laboratories and Forensic Medicine, Jordan University Hospital, Amman 11942, Jordan

**Keywords:** patient acceptance of health care, health knowledge, attitudes, practice, surveys and questionnaires, measles vaccine, parents

## Abstract

Measles remains a highly contagious and potentially severe infectious disease, necessitating high vaccine coverage. However, misinformation and measles vaccine hesitancy/resistance have posed significant challenges to achieving this goal. The COVID-19 pandemic further exacerbated these challenges, leading to a measles outbreak in Jordan in 2023. This study aimed to investigate the acceptance of the measles rubella (MR) vaccine among parents in Jordan and to identify its associated determinants. This cross-sectional questionnaire-based study was conducted using a previously Arabic-validated version of the Parental Attitudes towards Childhood Vaccines (PACV) survey instrument. Data collection took place in October 2023, and the final study sample comprised a total of 391 parents, with mothers representing 69.8% of the participants (*n* = 273). The majority of participating parents expressed either resistance (*n* = 169, 43.2%) or hesitancy (*n* = 168, 43.0%) towards MR vaccination, while only 54 participants (13.8%) expressed MR vaccine acceptance. Multivariate analysis revealed that trust in vaccine safety/efficacy, behavior, and having fewer offspring were significantly associated with MR vaccine acceptance. The current study revealed a concerning level of MR vaccine hesitancy/resistance among parents in Jordan, which could signal a public health alarm in the country. Urgent and targeted interventions are strongly recommended to address this issue, including mass campaigns aimed at building trust in the MR vaccine’s safety/efficacy. Additionally, there is an urgent need for effective public health initiatives to ensure sufficient measles vaccine coverage to prevent future outbreaks of this serious disease.

## 1. Introduction

Measles is a serious, highly contagious childhood infection, with significant levels of morbidity and mortality [1,2,3]. Its complications are attributable to the pathogenic effects of the measles virus, including severe pneumonia and central nervous system (CNS) complications, among others [4]. Rubella (German measles)—albeit less severe—is another viral exanthem that could have serious consequences if acquired by pregnant women, since congenital rubella syndrome can cause cardiac defects, deafness, and cataracts [5].

Since the 1960s, effective, safe, and affordable vaccination against measles and rubella has been available in the form of trivalent measles mumps rubella (MMR) vaccination [6]. The success of measles vaccination was manifested in the sharp decline in the number of measles cases, with its elimination in the United States in 2000 and with subsequent limited spread mostly due to imports or among pockets of unvaccinated individuals [7,8,9]. Despite the aspiration to eliminate measles, the disease is still endemic in many regions, mainly due to low vaccine coverage [10,11].

These safe and effective live attenuated measles-containing vaccines given in two doses for children at 9 and 15 months as MMR or MR formulations have been shown to be highly safe and effective [12]. However, outbreaks continue to occur mainly due to suboptimal vaccination coverage despite the reduction in the circulation of the wild-type measles virus [13,14,15]. The high rate of vaccination coverage is particularly important in the case of measles given its high basic reproductive number in the range of 12 to 18, rendering the level of population immunity needed for protection to exceed 95% [16].

One of the major reasons behind suboptimal measles vaccine coverage is parental vaccine hesitancy [17,18]. Factors driving parental measles vaccination hesitancy was recently systematically reviewed by Novilla et al. and included parental concerns and philosophical, moral, and religious objections [19].

Recent evidence showed that the coronavirus disease (COVID-19) pandemic had a profound negative impact on childhood vaccination coverage, leading to notable deficits [20,21,22,23]. Disruptions in vaccination coverage are known to be associated with an increase in vaccine-preventable disease (VPD) outbreaks [24]. To mitigate the risk of these outbreaks, a strategic intervention known as “catch-up vaccination” is strongly recommended [25].

In Jordan, a Middle Eastern Arab country, the National Expanded Programme on Immunization (EPI), led by the Jordanian Ministry of Health (MoH), has been at the forefront of public health efforts since its introduction in 1979. Since the establishment of mandatory routine childhood immunization in the country, notable achievements have been reported in Jordan, including the substantial control of several infectious diseases (e.g., Jordan has been polio free since 1992) [26,27].

The disruption of the Jordanian EPI during the COVID-19 pandemic had concerning consequences, which was manifested in an outbreak of measles in 2023, resulting in over 160 reported cases [28]. In response, the Jordanian MoH initiated a comprehensive measles rubella (MR) vaccination campaign targeting students in schools, kindergartens, orphanages, and events, commencing in mid-October 2023 [29]. Despite the public health importance of such an effort, this MR vaccination campaign faced immediate controversy within the Jordanian community due to the dissemination of recordings containing inaccurate and discouraging information about the MR vaccine [30,31].

In response to this misinformation, the Jordanian MoH promptly addressed the issue, declaring the videos as false and initiating legal actions against their spread, with clear emphasis on the MR vaccine’s proven effectiveness, rigorous approval procedures, and distribution readiness in Jordan [29].

Given the significance of this MR catch-up vaccination campaign and the need to address MR vaccine hesitancy, this study aimed to investigate the prevalence of parental hesitancy/resistance toward the MR vaccine. In addition, the study aimed to identify the underlying factors influencing parental vaccination hesitancy/resistance, utilizing a validated survey instrument. These aims could be crucial in understanding MR vaccine hesitancy and ensuring the success of the vaccination campaign in the country.

## 2. Materials and Methods

### 2.1. Study Design

This cross-sectional study employed an electronic questionnaire, which was based on the validated Parent Attitudes about Childhood Vaccines (PACV) instrument, adapted and validated in the Arabic language, reflecting the official language spoken in Jordan [32]. The survey instrument was adapted from a previous study by ElSayed et al., with slight modifications to tailor it specifically to MR vaccination [32].

The sampling approach chosen was convenience-based due to the time constraints and the urgent nature of the study, necessitating an efficient understanding of parental attitudes and perceptions regarding MR vaccination.

The calculation of the minimum sample size was conducted as follows: the Jordanian population was estimated to be 11,000,000 in 2023. The margin of error was set at 0.05, and the estimated proportion of interest (the prevalence of MR vaccine acceptance) was set at 0.5, given the uncertainty about the expected proportion. Finally, a confidence level of 95% was selected. Using the sample size formula for estimating a proportion and the aforementioned parameters, the minimum sample size was estimated at 385 participants [33].

The survey was accessible through Google Forms and was available for responses from 5 October to 11 October 2023, and the survey link was distributed using WhatsApp, Facebook, and Messenger. Participation was voluntary, without incentives for participation.

### 2.2. Ethical Aspects

The study was conducted in accordance with the Declaration of Helsinki and approved by the Institutional Review Board of The Faculty of Pharmacy at Applied Science Private University (Approval Number 2023-PHA-38 on 3 October 2023). Informed consent was a pre-requisite for participation, with a statement indicating the voluntary nature of participation and an acknowledgment that participants had read and understood the study information. The confidentiality of data was assured as well.

### 2.3. Description of the Questionnaire

The introductory section emphasized the inclusion criteria, including participants being adults aged 18 years or older, parents of children less than 15 years old, current residents in Jordan, and having the ability to read and understand the Arabic language.

Following the informed consent, “If you proceed to fill out the questionnaire, this indicates that you have decided to volunteer as a participant in this study, and that you have read and understood the information above”, the demographics of the participant were assessed.

The demographic variables included (1) age as a scale variable; (2) sex (male vs. female); (3) number of offspring as a scale variable; (4) educational level (high school or less vs. undergraduate vs. postgraduate); (5) governorate (Central: the Capital Amman, Balqa, Zarqa, or Madaba vs. North: Irbid, Jerash, Ajloun, or Mafraq vs. South: Karak, Maan, Tafilah, or Aqaba); (6) occupation (employed as a health care worker (HCW) vs. employed as non-HCW vs. unemployed/retired); (7) history of chronic disease (yes vs. not sure vs. no); (8) health insurance (yes vs. no); (9) monthly income of household (less than or equal to JOD (Jordanian dinar) 1000 vs. more than JOD 1000); (10) history of COVID-19 vaccine uptake, including the number of doses received; and (11) history of influenza vaccine uptake ever (yes vs. not sure vs. no).

In addition, the following questions were asked: (12) has your child(ren) received routine childhood immunizations listed by the MoH? (yes vs. not sure vs. no); (13) has your child(ren) received vaccines other than those that are part of the routine childhood immunizations listed by the MoH? (yes vs. not sure vs. no); and (14) to your knowledge, has your child(ren) previously received the MR vaccine? (yes vs. not sure vs. no).

### 2.4. PACV Items

The PACV scale employed in this study consisted of 14 items, each assessed using a 4-point Likert scale ranging from “always” to “often”, “sometimes”, and “never”. 

One of the PACV items aimed to evaluate parental MR vaccination hesitancy. Specifically, this item stated: “In general, to what extent do you consider yourself hesitant about the MR vaccine for children?” with responses categorized into three distinct groups as follows: “always” indicated MR vaccine resistance, “never” indicated MR vaccine acceptance), and “sometimes/often” indicated MR vaccine hesitancy.

The remaining PACV items were (1) have you ever postponed your child vaccination for reasons other than illness or allergies?; (2) have you ever decided to decline vaccinating your child for reasons other than illness or allergies?; (3) how sure are you that adhering to the recommended vaccination doses is beneficial for your child?; (4) children receive more vaccination doses than they need; (5) I believe that the MR vaccine protects against severe diseases; (6) it is better for my child to gain immunity by getting measles or rubella than by getting vaccinated; (7) it is best for children to get a few doses of vaccines at the same time; (8) how concerned are you that the MR vaccine will cause a serious side effect for your child?; (9) to what extent are you concerned that the MR vaccine for children is unsafe?; (10) how concerned are you that the MR vaccine will not protect against the disease?; (11) in general, to what extent do you consider yourself hesitant about the MR vaccine for children?; (12) I trust the information I receive about the MR vaccine; (13) I am able to discuss my concerns about the MR vaccine with my pediatrician; (14) in general, how much do you trust your pediatrician?

The internal consistency of the PACV scale was assessed using Cronbach α, with a calculated value of 0.745 indicating acceptable internal consistency.

### 2.5. Data Analysis

Analyses were conducted through IBM SPSS Statistics for Windows, Version 26.0. Armonk, NY: IBM Corp.

For the assessment of COVID-19 vaccine uptake, a scale was established based on the number of doses received: 0 doses, 1 dose, 2 doses, or 3 doses. Participants’ responses regarding influenza vaccine uptake were categorized as “none” (scored as 0), “not sure” (scored as 1), or “yes” (scored as 2). These two scores were then summed up for each participant. Those who obtained a total score of 2 or less were classified as having less favorable vaccine uptake history, while those with a score of 3 or more were categorized as having a favorable history of vaccine uptake.

In addition, previous child(ren) vaccine uptake was assessed using three items: the uptake of routine childhood immunizations, the uptake of non-routine childhood immunizations vaccines, and the self-reported history of previous MR vaccination for children. Each of these items was scored (“none” (scored as 0), “not sure” (scored as 1), or “yes” (scored as 2)), and the scores for all three items were summed up for each participant. Those who achieved a total score of 3 or less were considered to have less uptake of childhood vaccination, while those with a score of 4 or more were categorized as having more vaccine uptake.

For the PACV scale, a reversal of scores was implemented for items reflecting negative attitudes. Specifically, the Likert scale values were assigned as follows: “always” received a score of 0, “often” received a score of 1, “sometimes” received a score of 2, and “never” received a score of 3. This reversal allowed for the consistent interpretation of scores across the PACV items.

Associations between categorical variables were assessed using the chi-squared test, while associations between categorical and scale variables were conducted using the Mann–Whitney or Kruskal–Wallis tests, with *p* < 0.200 as the cut-off for incorporation in the multivariate analysis, which was carried out using multinomial logistic regression. The statistical significance was set at a cut-off of *p* < 0.050.

## 3. Results

### 3.1. Study Sample

A total of 435 responses were retrieved, of which 391 consented to participate (89.9%), and 44 individuals (10.1%) responded “no” to the mandatory informed consent item. The general features of the study sample are shown in (Table 1).

### 3.2. Parental MR Vaccination Hesitancy and Its Associated Factors

The majority of the participating parents were either resistant (*n* = 169, 43.2%) or hesitant (*n* = 168, 43.0%) towards MR vaccination. The factors associated with MR vaccination acceptance, which only was expressed by 54 participants (13.8%), are shown in Table 2.

In multivariate analysis, the only factors that were found to be significantly associated with MR vaccination acceptance (as opposed to hesitancy/resistance) were the number of offspring, previous behavior, and the perceived vaccine safety/efficacy (Table 3).

### 3.3. Factors Associated with PACV Constructs

Table 4 presents an in-depth analysis of the factors associated with the three constructs derived from the PACV scale.

The number of offspring significantly influenced the confidence in safety and efficacy construct (*p* = 0.021). Parents with fewer offspring had more confidence in the safety and efficacy of vaccination. Parental educational level played a significant role in the behavior construct (*p* = 0.014). Parents with a postgraduate level of education exhibited less favorable past behavior regarding childhood vaccination compared to those with undergraduate or high school or less level of education.

Additionally, the participating parents residing in the Central governorate exhibited greater levels of trust in vaccination (*p* = 0.005). Parents with a history of chronic disease, or those who were unsure about their history, exhibited more favorable behaviors (*p* = 0.001) compared to those with no history of chronic disease. Having health insurance showed a significant association with the more confidence in safety and efficacy construct (*p* = 0.041) and less trust (*p* = 0.045).

**Figure 1:**
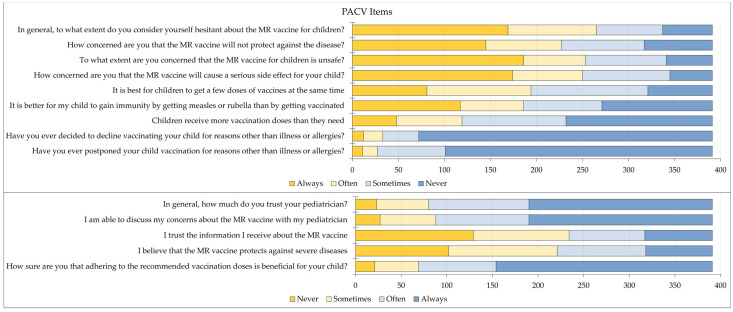
Responses of the participants for the 14 Parent Attitudes about Childhood Vaccines (PACV) items. MR: measles rubella.

The full responses of the participating parents for the 14 PACV items are presented in Figure 1.

## 4. Discussion

The current study was conducted swiftly amid the intense debate and controversy within the Jordanian community regarding the Jordanian MoH catch-up vaccination campaign for measles and rubella [29]. This initiative gained momentum due to the emergence of a measles outbreak that was reported in Jordan in 2023, resulting in over 160 reported cases [28]. This measles outbreak appeared to be linked to lapses in vaccination coverage, a phenomenon exacerbated by the disruptions caused by the COVID-19 pandemic, which was also reported in Jordan in a recent study by Abu-Rish et al. [28,34]. Additionally, it is worth noting that Jordan stands as one of the leading countries in terms of hosting refugees per capita. Within this vulnerable population, significant gaps in vaccination coverage exist, presenting a unique challenge in maintaining public health and preventing the resurgence of VPDs [35]. Furthermore, past and recent evidence from Jordan showed sub-optimal population immunity levels against measles [36,37]. Therefore, the MR catch-up vaccination campaign was a critical measure since measles is a highly contagious disease with the potential for severe complications, particularly among unvaccinated individuals [38,39].

The major finding of this study was the remarkably high level of parental hesitancy/resistance towards the MR vaccine, which exceeded 86%. This finding is both noteworthy and somewhat surprising, considering the context of Jordan’s previous achievements in childhood immunization and the public acceptance and embrace of mandatory childhood vaccination [40]. Historically, Jordan achieved considerable success in the EPI, which has been in place since 1979 [26]. This program has consistently achieved high vaccination coverage rates, leading to a substantial reduction in the incidence of infectious disease outbreaks in the country. The significant decrease in the prevalence of VPDs, such as measles and polio, has been a demonstration of the effectiveness of the Jordanian EPI [26].

One potential explanation for the notable finding of very high levels of MR vaccine hesitancy/resistance is the widespread dissemination of misinformation and the circulation of unsubstantiated conspiracy theories related to vaccination, which reached heightened levels during the COVID-19 pandemic [41,42,43]. The COVID-19 pandemic was accompanied by an infodemic—an overwhelming surge of misinformation—regarding the virus, preventive measures, and vaccines [44]. The infodemic created a suitable milieu for the proliferation of vaccine-related conspiracy ideas that were fueled by fear, mistrust, and uncertainty [45,46]. Misinformation and the spread of conspiracy ideas on social media platforms further exacerbated this issue, undermining the public confidence in vaccines in general [31,47]. In Jordan, previous studies conducted across various demographic strata demonstrated the pervasiveness of vaccination hesitancy and its close association with conspiracy beliefs [41,48,49]. These recent studies showed that a substantial portion of the population endorsed conspiratorial ideas regarding the intentions of vaccination programs and the safety of vaccines [41].

Of particular concern is the potential spillover effect of vaccine-related conspiracy beliefs into other emerging infectious diseases. Recent studies involving diseases like monkeypox have shown the wide embrace of such conspiratorial ideas, which can impact the willingness of people to accept vaccines [50,51]. This suggests that the consequences of vaccine hesitancy, driven by misinformation and conspiracy theories, extend beyond a single disease and can have broader implications for public health [52].

To contextualize the findings of the current study, it is essential to consider the broader perspective of research on parental attitudes towards measles vaccination worldwide. Comparing the results of this study to previous investigations revealed the alarmingly high rates of MR vaccine hesitancy/resistance observed in Jordan, as evidenced in the findings of this study. For example, a study from Ireland using the same survey instrument used in this study, namely PACV, reported a substantially lower prevalence of parental vaccine hesitancy. Among the 105 parents who participated in the survey, only 6.7% (7/105) exhibited hesitancy towards childhood vaccination [53]. This result appeared in sharp contrast with the findings of the current study in Jordan, where the prevalence of MR vaccine hesitancy exceeded 86%.

Similarly, a recent study from Saudi Arabia revealed a notably lower rate of vaccine hesitancy, with only 11% of parents expressing hesitancy towards childhood vaccination [54]. In Sudan, another study employing the PACV tool reported a vaccine hesitancy rate of 20%, much lower than the rate reported in the current study [55]. This substantial disparity in vaccine hesitancy rates should be taken in the context of the time, place, cultural, and contextual specificities of this alarming health issue [56,57]. 

In this study, a thorough investigation of the determinants of parental MR vaccine acceptance using the validated PACV tool highlighted several critical factors, similar to the recent systematic reviews on parental attitudes towards measles vaccination [18,19]. Among these factors, confidence in vaccine safety and efficacy emerged as a key element influencing the likelihood of parental MR vaccine acceptance [58,59]. 

One of the key aspects of confidence in vaccine safety is related to parental concerns about the number of doses their child receives [19]. Additionally, some participating parents in this study (48%) expressed a preference for natural immunity over MR vaccination, believing it to be a safer option. These concerns could be viewed as an overall reflection of the parental compromised trust in the safety of vaccines. Addressing these concerns necessitates a multifaceted approach, including the provision of clear yet simple, evidence-based information on the safety of the recommended vaccine schedule [60]. Public health campaigns are recommended to focus on educating parents about the proven effectiveness of the MR vaccine, particularly emphasizing its robust protection against the severe consequences of measles and its rapid spread in communities [61,62]. To encourage confidence in vaccine safety and efficacy, it is essential to clarify that vaccines offer a safe and effective means of acquiring immunity without the risks associated with natural infection [63,64]. Additionally, communication efforts should prioritize transparency about vaccine safety data and the extremely low risk of severe adverse events associated with MR vaccination [65,66].

In the context of this study, the behavior construct showed statistically significant differences concerning parental MR vaccine acceptance. The behavior construct represents a range of attitudes and behaviors related to vaccine decision making, reflecting the parental approach to vaccinating their children [67]. Health behavior is inter-related, resulting in individuals with consistent adherence to recommended vaccination schedules for themselves or their children typically demonstrating a pattern of making responsible health decisions [51,68]. Therefore, it is conceivable to consider that this pattern extends to the acceptance of the MR vaccine, as highlighted by the results of the current study. Thus, public health campaigns can benefit from improving health literacy and expanding access to health care services, subsequently enhancing vaccination acceptance rates [69].

In this study, further dissection of the factors associated with MR vaccine acceptance showed that individuals with postgraduate education exhibited lower scores in the behavior construct of PACV. This observation may suggest that higher levels of education might lead to more cautious attitudes towards vaccination, despite the difficulty to establish causality of this correlation [70]. However, further studies are needed to investigate this finding due to contradictory results in the literature on hesitancy towards childhood vaccination in association with parental education [71,72,73]. The study results also showed that parents with more offspring expressed less confidence in the safety and efficacy of the MR vaccine. A possible explanation of this result could be related to the diversity in past childhood vaccination experiences that comes with a higher number of children.

Residents in the Central region of Jordan, which harbors more than two-thirds of the entire population of the country, demonstrated higher levels of trust in MR vaccination. This result can be attributed to better access to health care services and information sources, which can contribute to increased trust in vaccination programs. 

Finally, the results of this study showed that parents with a history of chronic disease exhibited slightly more favorable behavior towards MR vaccination. This could be due to their heightened awareness of the importance of vaccination in preventing complications associated with chronic conditions [74].

### 4.1. Study Limitations

This current study results could provide valuable insights into parental attitudes towards MR vaccination in Jordan, especially within the context of an ongoing controversy regarding this vaccination campaign. However, it is crucial to recognize and acknowledge the study limitations upon interpreting and applying the findings in public health strategies and decision making. These limitations were as follows: (1) The cross-sectional design, while efficient for capturing the parental attitudes towards MR vaccination in Jordan, holds inherent limitations, including the incapability of establishing causal relationships or tracing the dynamic evolution of attitudes over time. (2) It is important to acknowledge that the selection of the convenience sampling approach introduces the possibility of selection bias. Therefore, caution should be implemented when extrapolating the study findings to the wider Jordanian population. (3) While the calculated sample size sufficed for the specific objectives of the study, it is important to recognize that the sample size was relatively small; therefore, the study findings can be regarded as preliminary, and a future extensive investigation with a larger sample size is necessary to meticulously assess the determinants of parental MR vaccine acceptance in Jordan. (4) The possibility of social desirability bias should be considered as well, since the participants might have been inclined to provide socially acceptable responses rather than responses reflecting their true attitudes towards MR vaccination. (5) Recall and reporting biases should be taken into consideration in the interpretation of the study findings since the parental recollection of past vaccination experiences might not be entirely accurate, in addition to the possibility of reporting bias if the parents provided answers that align with the societal norms. (6) The limited availability of previous literature and historical data on MR vaccine acceptance rates among Jordanian parents posed a challenge in assessing the influence of the COVID-19 pandemic on the attitude towards childhood vaccination.

### 4.2. Recommendations and Future Perspectives

Addressing the challenges of vaccine hesitancy and improving vaccine acceptance, particularly for the MR vaccine, require a multifaceted approach. One critical aspect is the necessity for clear and targeted public health campaigns that emphasize both the risks associated with diseases like measles and the safety and efficacy of their respective vaccines. To address this issue effectively, public health authorities are recommended to engage in proactive communication efforts. These efforts should aim to counteract the spread of misinformation by providing clear, readily accessible, evidence-based information about vaccines, with a specific focus on the MR vaccine.

In addition to disseminating accurate information, it is highly important to re-establish the trust and transparency within domestic vaccine programs. Building trust involves providing trustworthy information and the open, transparent sharing of data on the safety and efficacy of a vaccine, in addition to addressing concerns or questions from the public. This can be achieved through various channels, including health care providers, community leaders, and digital platforms, to ensure that reliable information is accessible in the various strata of Jordanian society.

Future studies are recommended with the inclusion of a comprehensive demographic analysis for better delineation of the factors influencing parental MR vaccine hesitancy. Specifically, this entails the need to distinguish between participants from urban and rural areas, given the potential variation in vaccination attitudes based on locality [75,76].

In light of the study’s findings, we propose the following specific evidence-based strategies to address parental MR vaccination hesitancy in Jordan: (1) The implementation of community-based initiatives involving HCWs, media figures, and influencers to promote MR vaccination. These initiatives should be designed based on established behavior change models and theories to establish trust in the safety of childhood immunization [77,78]. (2) The development of culturally appropriate educational materials that systematically address the prevalent vaccination concerns within the Jordanian community, and the use of various media channels, including social media, to disseminate these materials effectively [79,80,81]. (3) Empowering HCWs by training them in effective communication techniques to address vaccine hesitancy through active listening to parental concerns and providing evidence-based vaccine information [82,83,84]. (4) Continuous monitoring and research using valid methods to track the dynamics of parental vaccine hesitancy to anticipate and address evolving concerns promptly through data-driven interventions [85,86]. (5) Establishing stronger partnerships with international health agencies to strengthen the Jordanian expertise and resources in tackling vaccine hesitancy [87,88].

## 5. Conclusions

The substantial disparity in vaccine hesitancy rates between previous studies on parental attitude to measles vaccination and the results of the current study in Jordan highlights the severity of the situation in the country. While vaccine hesitancy is a multifaceted, complex issue influenced by numerous factors, the exceptionally high rates of MR vaccination hesitancy/resistance observed in this study is of particular concern. This highlights the urgent need for tailored interventions specifically designed to address the unique emergent challenge of parental hesitancy towards MR vaccination in Jordan.

The study findings could be viewed as useful insights into the possible factors that could influence parental acceptance of the MR vaccine. Besides the confidence in vaccine safety/efficacy and behaviors, the family size, educational level, place of residence, and chronic disease history were possible variables associated with parental attitudes towards MR vaccination. Recognizing these factors and their individual contributions is essential for designing effective strategies to promote MR vaccine acceptance in Jordan.

The future of vaccine acceptance, particularly in the context of MR vaccination, relies on the implementation of comprehensive and targeted communication strategies. These strategies can benefit from emphasizing the safety and efficacy of MR vaccination as well as from combating the spread of misinformation, particularly on social media platforms. The establishment of trust and transparency within vaccine programs is also of particular importance. By addressing these critical issues, public health authorities can achieve improved MR vaccine coverage, with a subsequent reduction in disease burden, to achieve better public health outcomes. The urgent issue of MR vaccination hesitancy/resistance in Jordan cannot be overstated, as it is pertinent to the global efforts aiming to address vaccine hesitancy and to protect communities from serious VPDs.

## Figures and Tables

**Table 1:** General features of the study sample (*n* = 391).

Variable	Category	N ^5^ (%)
Age	<40 years	213 (54.5)
	≥40 years	178 (45.5)
Sex	Male	118 (30.2)
	Female	273 (69.8)
Offspring	1 or 2	173 (44.2)
	3 or more	218 (55.8)
Educational level	High school or less	97 (24.8)
	Undergraduate	245 (62.7)
	Postgraduate	49 (12.5)
Governorate	Center	265 (67.8)
	North	98 (25.1)
	South	28 (7.2)
Occupation	Employed (HCW ^3^)	52 (13.3)
	Employed (non-HCW)	171 (43.7)
	Unemployed	168 (43.0)
History of chronic disease	Yes/not sure	104 (26.6)
	No	287 (73.4)
Health insurance	Yes	319 (81.6)
	No	72 (18.4)
Monthly income of household	JOD ≤ 1000 ^4^	315 (80.6)
	JOD > 1000	76 (19.4)
Parental COVID-19 ^1^ vaccine uptake	None/single dose	62 (15.9)
	Primary series	259 (66.2)
	Booster doses	70 (17.9)
Parental influenza vaccine uptake	None	230 (58.8)
	Not sure	45 (11.5)
	Yes	116 (29.7)
Uptake of routine childhood immunizations	None	12 (3.1)
	Not sure	8 (2.0)
	Yes	371 (94.9)
Uptake of non-routine childhood immunizations	None	325 (83.1)
	Not sure	16 (4.1)
	Yes	50 (12.8)
Child MR ^2^ vaccine uptake	None	180 (46.0)
	Not sure	61 (15.6)
	Yes	150 (38.4)

^1^ COVID-19: coronavirus disease 2019; ^2^ MR: measles rubella; ^3^ HCW: health care worker; ^4^ JOD: Jordanian dinar; ^5^ N: number.

**Table 2:** Factors associated with parental MR vaccination hesitancy.

Variable	Category	MR ^4^ Vaccine Acceptance		*p*, χ^2^
		Acceptance	Hesitancy/Resistance	
		N ^5^ (%)	N (%)	
Age	<40 years	34 (16.0)	179 (84.0)	0.177, 1.820
	≥40 years	20 (11.2)	158 (88.8)	
Sex	Male	20 (16.9)	98 (83.1)	0.237, 1.398
	Female	34 (12.5)	239 (87.5)	
Offspring	1 or 2	34 (19.7)	139 (80.3)	**0.003**, 8.898
	3 or more	20 (9.2)	198 (90.8)	
Educational level	High school or less	11 (11.3)	86 (88.7)	0.508, 1.353
	Undergraduate	34 (13.9)	211 (86.1)	
	Postgraduate	9 (18.4)	40 (81.6)	
Governorate	Center	41 (15.5)	224 (84.5)	0.385, 1.911
	North	10 (10.2)	88 (89.8)	
	South	3 (10.7)	25 (89.3)	
Occupation	Employed (HCW ^2^)	10 (19.2)	42 (80.8)	0.462, 1.544
	Employed (non-HCW)	23 (13.5)	148 (86.5)	
	Unemployed	21 (12.5)	147 (87.5)	
History of chronic disease	Yes/not sure	14 (13.5)	90 (86.5)	0.904, 0.015
	No	40 (13.9)	247 (86.1)	
Health insurance	Yes	47 (14.7)	272 (85.3)	0.266, 1.239
	No	7 (9.7)	65 (90.3)	
Monthly income of household	JOD ≤ 1000 ^3^	44 (14.0)	271 (86.0)	0.854, 0.034
	JOD > 1000	10 (13.2)	66 (86.8)	
Previous parent vaccination history score	<3	22 (10.4)	190 (89.6)	**0.032**, 4.586
	≥3	32 (17.9)	147 (82.1)	
Previous child vaccination history score	<4	25 (11.4)	194 (88.6)	0.121, 2.399
	≥4	29 (16.9)	143 (83.1)	
PACV score ^1^	≤23	3 (1.5)	200 (98.5)	**<0.001**, 53.948
	>23	51 (27.1)	137 (72.9)	
Behavior construct	≤8	19 (9.0)	193 (91.0)	**0.002**, 9.145
	>8	35 (19.6)	144 (80.4)	
Safety and efficacy construct	≤10	4 (1.8)	224 (98.2)	**<0.001**, 66.786
	>10	50 (30.7)	113 (69.3)	
Trust construct	≤6	25 (10.0)	225 (90.0)	**0.004**, 8.458
	>6	29 (20.6)	112 (79.4)	

^1^ PACV: Parent Attitudes about Childhood Vaccines; ^2^ HCW: health care worker; ^3^ JOD: Jordanian dinar; ^4^ MR: measles rubella; ^5^ N: number. Significant *p* values are highlighted in bold.

**Table 3:** Factors associated with MR vaccine acceptance in multinomial logistic regression analysis.

Model	aOR (95% CI) ^2^	*p*
**MR ^1^ Vaccine Acceptance vs. MR Vaccine Hesitancy/Rejection**		
**Nagelkerke R^2^ = 0.392**		
Age < 40 years	1.27 (0.60–2.69)	0.529
Age ≥ 40 years	Ref.	
Offspring 1 or 2	2.10 (1.01–4.34)	**0.046**
Offspring 3 or more	Ref.	
Previous parent vaccination history score < 3	0.62 (0.31–1.23)	0.174
Previous parent vaccination history score ≥ 3	Ref.	
Previous child vaccination history score < 4	0.51 (0.25–1.05)	0.068
Previous child vaccination history score ≥ 4	Ref.	
Behavior construct score > 8	2.62 (1.30–5.30)	**0.007**
Behavior construct score ≤ 8	Ref.	
Safety and efficacy construct score > 10	23.61 (8.14–68.4)	**<0.001**
Safety and efficacy construct score ≤ 10	Ref.	
Trust construct score > 6	1.40 (0.69–2.86)	0.350
Trust construct score ≤ 6	Ref.	

^1^ MR: measles rubella; ^2^ CI: confidence interval, aOR: adjusted odds ratio. Significant *p* values are highlighted in bold.

**Table 4:** Factors associated with PACV constructs.

Variable	Category	Behavior Construct		Confidence in Safety and Efficacy Construct		Trust Construct	
		Mean ± SD ^3^	*p*	Mean ± SD	*p*	Mean ± SD	*p*
Age	<40 years	7.8 ± 1.6	0.472	9.9 ± 4.9	0.219	5.8 ± 2.1	0.342
	≥40 years	7.6 ± 1.8		9.3 ± 4.8		5.6 ± 2.2	
Sex	Male	7.7 ± 1.8	0.718	10.1 ± 5	0.203	5.7 ± 2.2	0.879
	Female	7.7 ± 1.6		9.4 ± 4.8		5.8 ± 2.1	
Offspring	1 or 2	7.7 ± 1.8	0.993	10.3 ± 5.2	**0.021**	5.9 ± 2.2	0.196
	3 or more	7.8 ± 1.6		9.1 ± 4.5		5.6 ± 2.1	
Educational level	High school or less	7.7 ± 1.8	**0.014**	9 ± 4.8	0.343	5.7 ± 2.1	0.690
	Undergraduate	7.8 ± 1.5		9.9 ± 5		5.8 ± 2.2	
	Postgraduate	7.1 ± 1.9		9.4 ± 4.3		5.6 ± 2.0	
Governorate	Center	7.9 ± 1.6	0.066	9.8 ± 4.9	0.534	5.9 ± 2.1	**0.005**
	North	7.5 ± 1.8		9.5 ± 4.8		5.2 ± 2.0	
	South	7.4 ± 1.9		8.7 ± 5.2		5.5 ± 2.4	
Occupation	Employed (HCW ^1^)	7.9 ± 1.5	0.647	10.3 ± 5.0	0.481	5.7 ± 2.0	0.490
	Employed (non-HCW)	7.6 ± 1.8		9.6 ± 4.9		5.6 ± 2.1	
	Unemployed	7.8 ± 1.6		9.4 ± 4.8		5.8 ± 2.2	
History of chronic disease	Yes/not sure	8.1 ± 1.5	**0.001**	9.2 ± 4.8	0.257	6.0 ± 2.0	0.159
	No	7.6 ± 1.7		9.8 ± 4.9		5.6 ± 2.2	
Health insurance	Yes	7.7 ± 1.7	0.079	9.9 ± 4.8	**0.041**	5.6 ± 2.1	**0.045**
	No	8.0 ± 1.5		8.6 ± 4.9		6.2 ± 2.1	
Monthly income of household	JOD ≤ 1000 ^2^	7.8 ± 1.6	0.150	9.5 ± 4.9	0.485	5.8 ± 2.2	0.206
	JOD > 1000	7.5 ± 1.8		9.9 ± 4.6		5.5 ± 1.9	
Previous parent vaccination history score	<3	7.8 ± 1.5	0.631	9.3 ± 4.6	0.286	5.7 ± 2.0	0.299
	≥3	7.6 ± 1.8		10.0 ± 5.2		5.8 ± 2.2	
Previous child vaccination history score	<4	7.8 ± 1.8	0.059	9.4 ± 4.9	0.271	5.9 ± 2.1	0.162
	≥4	7.7 ± 1.5		9.9 ± 4.9		5.6 ± 2.2	

^1^ HCW: health care worker; ^2^ JOD: Jordanian dinar; ^3^ SD: standard deviation. *p* values were calculated using the Mann–Whitney or the Kruskal–Wallis tests. Significant *p* values are highlighted in bold.

## Data Availability

Data presented in the current study are available upon request from the corresponding author (M.S.).

## Abbreviations

aOR	Adjusted odds ratio
CI	Confidence interval
CNS	Central nervous system
COVID-19	Coronavirus disease 2019
EPI	The National Expanded Programme on Immunization
HCW	Health care worker
JOD	Jordanian dinar
MMR	Measles mumps rubella
MoH	Ministry of Health
MR	Measles rubella
PACV	Parent Attitudes about Childhood Vaccines
VPD	Vaccine-preventable disease
